# Effects of Scaling and Root Planing on Salivary Interleukine-6 Levels in Chronic Periodontitis Patients and Glycemic Controls

**DOI:** 10.7759/cureus.45388

**Published:** 2023-09-17

**Authors:** Sanghamitra Ghosh, Gayathri C Buyyanapragada, Neelam Gavali, Mohammad ismail, Ramnath Elangovan, Nch. Ramya Sri Lakshmi

**Affiliations:** 1 Periodontology, New Horizon Dental College and Research Institute, Bilaspur, IND; 2 Periodontology, AECS Maaruti College of Dental Sciences, Bengaluru, IND; 3 Periodontology, Bharati Vidyapeeth (deemed to be) University, Dental College and Hospital, Pune, IND; 4 Periodontology, Mithila Minority Dental College and Hospital, Darbhanga, IND; 5 Periodontology, School of Dentistry, University of Rwanda, Kigali, RWA; 6 Periodontology, MK Dental Clinic, Hyderabad, IND

**Keywords:** inflammatory marker, probing depth, type-2 diabetes mellitus, salivary il-6, chronic periodontitis

## Abstract

Background: Type 2 diabetes mellitus (DM) and periodontitis have a bidirectional relationship that is well documented in many reviews and epidemiological studies. Periodontitis has been referred to as the sixth complication of diabetes mellitus. Various studies showed improvement in Interleukin-6 levels as well as metabolic parameters after non-surgical periodontal therapy in chronic periodontitis patients with type 2 DM.

Objective: To evaluate the effect of scaling and root planing (SRP) on salivary levels of IL-6 and assessment of clinical parameters in CP patients with and without T2DM.

Methods: We included 50 CP patients with well-controlled T2DM (Group I), and 50 CP patients without T2DM as controls (Group II) with evident clinical inflammation, ≥ 5mm probing depth (PD) and a relative attachment level (RAL) of ≥ 5mm. Following a brief medical and dental history plaque index (PI), gingival index (GI), gingival bleeding index (BI), PD, and RAL were recorded, and an unstimulated saliva was collected. Following SRP therapy, the clinical parameters and IL-6 levels were measured after seven days, 14 days, and 30 days. Intragroup and intergroup comparisons were carried out using a paired t-test and an independent t-test. The statistical significance was set at P < 0.05. Data were analyzed using computer software, Statistical Package for Social Sciences (SPSS) v. 22.0 (IBM Corp., Armonk, NY).

Results: Intergroup comparisons of IL-6 levels at different intervals showed a significantly higher reduction in Group II than in Group I (p=0.000). While the mean difference in the GI scores from baseline to 30 days was significantly higher in Group I patients (p=0.000), the difference in the mean PI (p=0.004), mean BI (p=0.000), mean PD (p=0.000) and mean RAL scores (p=0.000) were significantly higher in Group II patients.

Conclusion: This study indicates that scaling and root planing is effective in glycemic control and also has a role to play in the level of salivary IL-6 in periodontal health and T2DM with chronic periodontitis. Elevated salivary IL-6 levels indicate periodontal inflammation which is further increased in T2DM patients. Hence, elevated IL-6 can be considered a marker of periodontal destruction.

## Introduction

Periodontitis is one of the most common diseases of humans, generally believed to be an inevitable consequence of aging. However, we have learned over time that not all people, nor all populations, are at equal risk of developing periodontitis [1]. Although primarily caused by a group of specific microorganisms, factors including smoking, genetics, and hormones increase the risk of periodontitis. The relationship between type 2 diabetes mellitus (T2DM) and periodontitis is well established [2]. Diabetic patients are at increased risk of developing periodontitis, and severity increases in patients with poor diabetic control [3,4]. Moreover, the presence of circulating cytokines in DM increases the risk of infections, especially periodontitis [5]. Glycemic control and control of periodontal infection run hand in hand. Along with reducing local inflammation, effective management of periodontal infection leads to a reduction in glycated hemoglobin levels in DM patients. Likewise, good and moderate glycemic control helps in controlling the periodontal infection [6].

The inflammatory response to periodontal infection is characterized by an increased release of inflammatory mediators, including cytokines, chemokines, and C-reactive proteins (CRP) [7]. Interleukin 6 (IL-6) is a pleiotropic protein with a range of activities, including neutrophil differentiation during infection and protection against septic shock, and tumorigenic and tumorostatic activities [8]. Previous studies have suggested the presence of elevated levels of salivary IL-6 at the site of periodontal infection, especially in diabetic patients, confirming the influence of DM on periodontitis [9,10]. Hence, salivary IL-6 is considered an important biomarker to predict the prognosis of periodontitis.

Initial non-surgical treatment of periodontitis with scaling and root planing (SRP) substantially reduces the clinical inflammation, probing depth (PD), bleeding on probing (BOP), and further clinical attachment level (CAL) gain. Furthermore, it is associated with a reduction in salivary IL-6 levels [11]. Similarly, the benefits of SRP in diabetic patients are two-fold. Reduction in clinical inflammation and salivary IL-6 levels help in the metabolic control of diabetes [12]. With this understanding, we conducted the present study to evaluate the effect of SRP on salivary levels of IL-6 and clinical parameters in CP patients with and without T2DM.

## Materials and methods

This clinico-biochemical study includes 100 chronic periodontitis patients (51 female and 49 male) aged between 30-60 years chosen based on the formula n = (2 * sigma^2 / delta^2) * (Z_alpha/2 + Z_beta)^ 2 where n = required sample size per group (chronic periodontitis and glycemic controls), δ = minimum clinically significant difference in IL-6 levels between the two groups who visited the out-patient department of Periodontology, New Horizon Dental College and Research Institute, Bilaspur. We obtained approval from the Institutional Ethics Committee (NHDCRI/2018/MDS/PERI/06-ECC) and informed consent was achieved from the patients prior to the study. We included 50 CP patients with well-controlled T2DM with a glycated hemoglobin level of 6-8%, diagnosed at least three years prior to the study (Group I), and 50 CP patients without T2DM as controls (Group II). Criteria for inclusion were the presence of at least 20 natural teeth, evident clinical inflammation with ≥ 5mm probing depth (PD) in 30% of sites, and a relative attachment level (RAL) of ≥ 5mm. We excluded patients with systemic diseases including, epilepsy, hypertension, asthma, thyroid, autoimmune diseases, and cardiac diseases; patients on antibiotic therapy in the past three months and immunosuppressive therapy; history of periodontal therapy in the past six months; lactating and pregnant mothers and those with habits including smoking and chewing tobacco.

**Figure 1:**
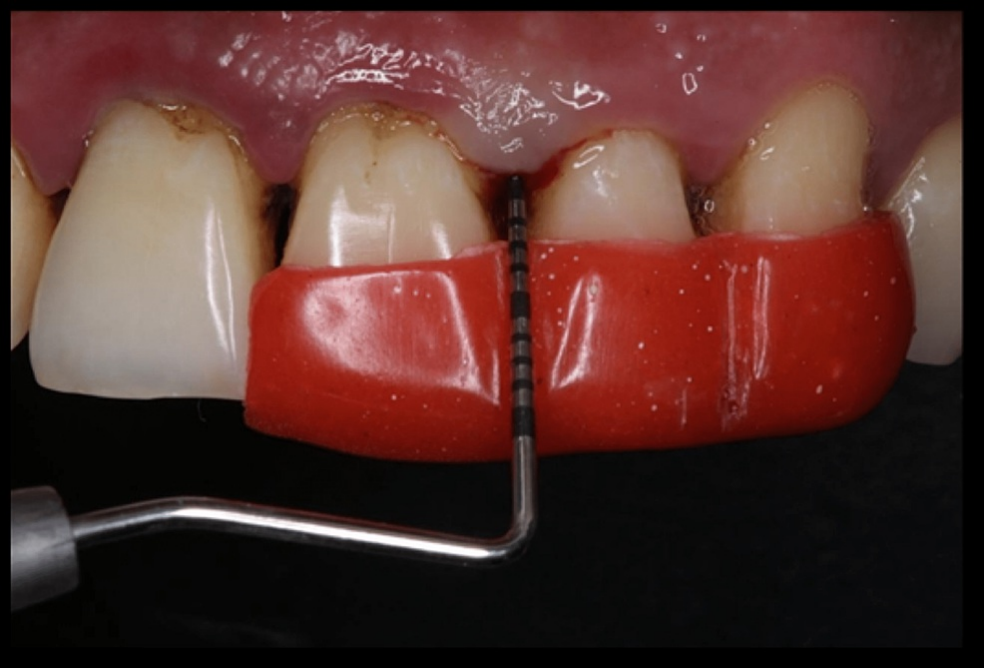
Measurement of Relative Attachment Level Using Stent

All patients underwent SRP following a brief medical and dental history recording and a complete and detailed baseline clinical examination using a sterile mouth mirror, and a UNC-15 graded periodontal probe was performed. Measurement of clinical parameters including plaque index (PI), gingival index (GI), gingival bleeding index (BI), pocket depth (PD), and relative attachment level (RAL) were recorded by a single examiner at baseline (before SRP), after 7 days, 14 days, and 30 days following SRP therapy (Figure 1). A single observer was also employed to avoid bias in the study. An unstimulated saliva sample was collected at baseline, after 7 days, 14 days, and 30 days following SRP therapy, and subjected to enzyme-linked immunosorbent assay (ELISA) to assess salivary IL-6 levels (Figure 3). All recorded data entered in Microsoft Excel was imported to SPSS version 22.0 for statistical analysis. Continuous variables such as plaque index, gingival index, gingival bleeding index, pocket depth, and relative attachment level were expressed as mean ± standard deviation and categorical variables were expressed as frequency and percentages. Intragroup and intergroup comparisons were carried out using a paired t-test and an independent t-test, respectively. A p-value of <0.05 was considered statistically significant.

**Figure 2:**
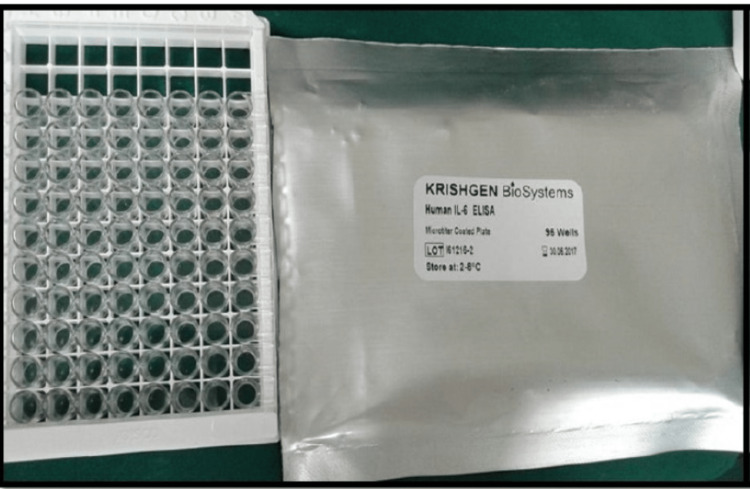
Prepared Wells for ELISA Test

## Results

The study cohort consisted of 51 female and 49 male patients with a mean age of 42.3±8.59 years. Table 1 summarizes the intragroup comparisons of the clinical parameters, including PI, GI, BI, PD RAL, and IL-6 levels. In Group I, there was a significant reduction in the mean IL-6 levels from baseline (152.54±1.46 pg/μL) to 7 days (144.74±2.67pg/μL), 14 days (130.10±2.74pg/μL) and 30 days (117.49±1.50pg/μL) after SRP (p=0.000). A similar reduction was observed in the mean IL-6 levels baseline (135.34±3.01pg/μL) to 7 days (120.32±2.52pg/μL), 14 days (103.69±2.08pg/μL), and 30 days (82.33±1.42pg/μL) after treatment in Group II patients (p=0.000). Intergroup comparisons of IL-6 levels at different intervals showed a significantly higher reduction in Group II than in Group I (p=0.000).

**Table 1:** Intragroup comparisons of clinical parameters and Il 6 levels in the study groups

	Duration	Baseline	7 days	14 days	30 days	P-value
Group I	Plaque index	3.77± 0.41	3.18 ±0.15	2.51±0.31	1.95±0.36	0.000
	Gingival index	1.66± 0.19	1.26 ± 0.15	0.54 ± 0.19	0.35± 0.08	0.000
	Bleeding index	85.96 ±2.43	75.07±2.92	62.51± 1.48	52.54± 1.53	0.000
	Probing depth	5.28 ±0.77	4.71± 0.45	3.54 ±0.31	3.14 ±0.21	0.000
	Relative attachment level	14.00± 0.62	13.57 ±0.86	12.07± 0.59	10.01 ±0.59	0.000
	IL 6 levels	152.54±1.46	144.74±2.67	130.10±2.74	117.49±1.50	0.000
Group II	Plaque index	3.62± 0.31	3.13 ±0.30	2.43 ±0.26	1.50±0.29	0.000
	Gingival index	1.27 ±0.16	0.93 0.23	0.51± 0.25	0.31± 0.12	0.000
	Bleeding index	86.77 ±2.96	72.70 1.52	63.20± 1.74	47.52± 1.40	0.000
	Probing depth	5.51 ±0.79	4.47 0.28	3.40 ±0.26	2.44± 0.31	0.000
	Relative attachment level	15.08 ±0.56	13.04 ±0.59	12.03± 0.55	9.01± 0.55	0.000
	IL-6 levels	135.34±3.01	120.32±2.52	103.69±2.08	82.33±1.42	0.000

In Group I, significant improvement in mean GI was observed from the baseline (3.77± 0.41) to 7 days (3.18 ±0.15), 14 days (2.51±0.31), and 30 days (1.95±0.36) after treatment (p=0.000). Similar improvements were observed in the mean GI (1.66±0.19 vs. 0.35±0.08; p=0.000), mean BI (85.96±2.43 vs. 52.54±1.53; p=0.000), mean PD (5.28 ±0.77mm vs. 3.14±0.21 mm; p=0.000) and mean RAL (14.00±0.62mm vs. 10.01±0.59mm p=0.000) scores from baseline to 30 days after SRP. In Group II, the mean PI at baseline was 3.62±0.31, which improved to 3.13±0.30, 2.43±0.26, and 1.50±0.29 at 7, 14, and 30 days after SPR, respectively (p=0.000). Similar improvements were observed in the mean GI (p=0.000), mean BI (p=0.000), mean PD (p=0.000), and mean RAL scores (p=0.000) (Table 1).

Table 2 summarizes the intergroup comparisons of clinical parameters at different study intervals. The mean difference in the PI from baseline to 7 days (0.59±0.26 vs. 0.49±0.01, p=0.264) and baseline to 14 days (1.26±0.10 vs. 1.19±0.05, p=0.384) was similar in both groups, while the difference in the mean PI from baseline to 30 days was significantly higher in group II (1.82±0.05 vs.2.12±0.02, p=0.004). While the mean difference in the GI scores from baseline to 30 days was significantly higher in Group I patients (1.31±0.11 vs. 0.96±0.04, p=0.000), the mean difference in the BI (33.42±0.90 vs. 39.25±1.56, p=0.000), PD (2.14±0.56mm vs. 3.07±0.48mm, p=0.000) and RAL scores (3.99±0.03mm vs. 6.07±0.01mm, p=0.000) was significantly higher in Group II patients.

**Table 2:** Intergroup comparison of mean difference of clinical parameters at different intervals between two groups

Variable	Study intervals	Mean difference (Mean ± Standard deviation)		
		Group I	Group II	P value
Plaque index	Baseline to 7 days	0.59±0.26	0.49±0.01	0.264
	Baseline to 14 days	1.26±0.10	1.19±0.05	0.384
	Baseline to 30 days	1.82±0.05	2.12±0.02	0.004
Gingival index	Baseline to 7 days	0.40±0.04	0.34±0.07	0.250
	Baseline to 14 days	1.12±0.00	0.76±0.09	0.000
	Baseline to 30 days	1.31±0.11	0.96±0.04	0.000
Bleeding index	Baseline to 7 days	10.89±0.49	14.07±1.44	0.000
	Baseline to 14 days	23.45±0.95	23.57±1.22	0.840
	Baseline to 30 days	33.42±0.90	39.25±1.56	0.000
Probing depth	Baseline to 7 days	0.57±0.32	1.04±0.51	0.010
	Baseline to 14 days	1.74±0.46	2.11±0.53	0.023
	Baseline to 30 days	2.14±0.56	3.07±0.48	0.000
Relative attachment level	Baseline to 7 days	0.43±0.24	2.04±0.03	0.000
	Baseline to 14 days	1.93±0.03	3.05±0.01	0.000
	Baseline to 30 days	3.99±0.03	6.07±0.01	0.000

## Discussion

The persistence of host inflammatory response against microorganisms in periodontitis leads to the destruction of tooth-supporting structures [13]. The transition from acute to chronic inflammatory stages in chronic periodontitis is characterized by the accumulation and differentiation of immune cells at the site of inflammation, with a resultant elevation in the salivary IL-6 levels [14]. The literature suggests that persistent periodontal inflammation invariably increases the presence of inflammatory mediators that play a role in periodontal disease initiation, progression, and resolution of periodontitis [15,16]. On the other hand, with increased production of IL-6 in T2DM patients, they are prone to severe periodontal inflammation [17]. As IL-6 is now considered an inflammatory marker for chronic periodontitis, we evaluated the effect of SRP on the salivary IL-6 levels and clinical diagnostic parameters of periodontitis in T2DM patients and CP patients without DM. Considering the non-invasive procedure and ease of sample collection, saliva samples were preferred over serum and GCF to evaluate IL-6 levels [18]. Similarly, for the salivary analysis, ELISA was used due to its high sensitivity and specificity.

While Batool et al. [19] reported significantly elevated IL levels in CP patients as compared to healthy subjects, Balaji et al. [20] have reported elevated IL-6 in DM with CO compared to CP and DM patients. As suggested by Lima et al. [21] in a systematic review, we observed higher baseline IL-6 values in both groups. However, compared to Group II, the IL-6 was much higher in Group I patients (152.54±1.46 pg/μL vs.. 135.34±3.01pg/μL), suggesting the combined effect of altered immune response with DM and CP on IL levels. The IL-6 levels positively correlated with the severity of periodontitis, and the values decreased with the resolution of inflammation after periodontal therapy. Similar to Eivazi et al. [22] we observed a significant reduction in the IL-6 levels observed in individual groups; however, the mean difference in the IL-6 levels at different intervals was much lower in Group I. The findings confirm the role of pro-inflammatory cytokine levels in the pathogenesis of periodontitis and T2DM.

As IL-6 is associated with increased inflammation, PI, BI, and GI were used to comparatively evaluate the clinical inflammation and PD and RAL to assess the severity of disease before and after SRP in CP patients with or without T2DM. In our study, the mean baseline plaque scores of Groups I and II patients were comparable (3.77± 0.41 vs. 3.62 ±0.3). Following the SRP therapy, a significant reduction in the mean PI after 7 days, 14 days, and 30 days was observed in individual groups. The results are in accordance with Aziz et al. [12] and Talbert et al. [23] who observed a significant reduction in plaque score after SRP. The reduction in PI corresponds to the reduction in microbial deposits on the tooth surfaces and gingival crevices. Furthermore, oral hygiene instructions given at their therapy and increased self-awareness of being regularly checked for oral hygiene could have motivated the patients to clean their teeth surfaces regularly. 

Although many indices have been proposed to assess gingival health, due to the reliability, validity, and ease of recording, the GI introduced by Loe and Silness (1963) remains the preferred choice amongst practitioners. At baseline, the mean BI was comparable between groups, while the mean GI was slightly higher in Group I than in Group II. Higher BI is correlated to increased inflammation in DM patients. Similar to studies by Acharya et al. [24] and Agarwal et al. [25], significant reductions in the GI were noted in individual groups at baseline and in 6 months. Improved GI scores correspond to the reduction in plaque, calculus, and subsequent resolution of gingival inflammation. Also, the reduction in GI was significantly higher in Group I as compared to Group II. This corresponds to the effectiveness of SRP in reducing blood glucose levels and HbA1c levels and the corresponding local inflammatory response. [24,25]

PD and RAL are correlated to the clinical inflammation indices in periodontitis. Increased PD and RAL correspond to advanced periodontitis and are a characteristic feature in T2DM patients. The baseline mean PD (5.28±0.77mm vs. 5.51±0.79mm) and RAL (14±0.62mm vs. 15.08±0.56mm) were comparable between groups. Literature suggests the improvement in PD and RAL post-therapy. Jain et al. [26] and Miranda et al. [27] observed improved glycated hemoglobin levels and corresponding improvement in the PD and RAL after SRP. Similarly, we observed a significant reduction in PD and improved RAL post-SRP therapy. Intergroup comparisons showed more significant improvements in Group II, which correspond to the decreased levels of IL-6 with the resultant decrease in inflammatory mediators, which provides a favorable environment for tissue healing and new attachment formation at the tissue healing sites [18].

Limitations and recommendations

Although the positive effect of SRP on IL-6 levels in CP patients with or without T2DM is observed in our study, the study has some inherent limitations. Firstly, only well-controlled DM patients were included, which limited us in evaluating whether non-surgical treatment improves glycated hemoglobin levels. Secondly, the shorter follow-up of only 30 days might have biased the clinical indices. Awareness of the patient as being evaluated at regular short intervals might have led to conscious cleaning of teeth surfaces. Hence, further studies with longer are warranted to evaluate the effect of SRP on IL-6 and clinical parameters in patients with both controlled and uncontrolled T2DM. 

## Conclusions

The result of this study indicates that scaling and root planing is effective in glycemic control and also has a role to play regarding salivary IL-6 in periodontal health and type 2 diabetes mellitus with chronic periodontitis. However, further longitudinal studies with a larger sample size are required to confirm the impact of periodontal disease with diabetes on IL-6 levels with diabetes mellitus.
